# Targeting KRAS G12C in Rare Mesonephric Carcinoma of the Cervix: A Promising Response to Sotorasib

**DOI:** 10.1200/PO-25-01077

**Published:** 2026-05-06

**Authors:** Bryan Cho Wing Li, Joshua Jing Xi Li, Ka Yu Tse, Haonan Lu, Karen Kar Loen Chan

**Affiliations:** ^1^ Centre of Cancer Medicine, The University of Hong Kong, Hong Kong SAR, China; ^2^ Department of Pathology, The University of Hong Kong, Hong Kong SAR, China; ^3^ Department of Obstetrics and Gynaecology, The University of Hong Kong, Hong Kong SAR, China

## Case Presentation

A 52-year-old Chinese female presented with per vaginal spotting and was diagnosed with mesonephric-type cervical cancer (CA cervix), stage 3c1(p) pT2aN1, in January 2023. Histopathologic analysis revealed human epidermal growth factor receptor 2 immunohistochemistry score 2 (DISH-negative), human papillomavirus–negative, PD-L1–negative, and intact mismatch repair proteins. Next-generation sequencing (NGS) was performed on the original surgical sample at the time of recurrence and identified a *KRAS* G12C mutation.

Preoperative clinical and radiologic assessment indicated stage I disease. Consequently, the patient was subjected to radical hysterectomy with bilateral salpingo-oophorectomy and pelvic lymph node dissection. However, the final pathologic staging was upstaged to IIIC1(p) (pT2aN1) after the identification of two metastatic right pelvic lymph nodes. Therefore, adjuvant chemoradiotherapy together with cisplatin was administered.

The first recurrence was found in October 2023, with bilateral lung metastasis, pleural and peritoneal deposits, and mediastinal nodal metastasis. It was treated with six cycles of carboplatin, paclitaxel, and bevacizumab, achieving a metabolic remission. It was followed by maintenance bevacizumab. Pembrolizumab was not approved locally for first-line treatment at that time. Tisotumab vedotin was not available locally also. However, disease progression occurred in May 2024 with lung and bone metastases. Despite salvage therapy with carboplatin and gemcitabine, the disease progressed.

In July 2024, the patient developed brain metastases with confusion and underwent whole-brain radiotherapy. At this juncture, she was referred to our center for further management. Subsequent therapy with trastuzumab deruxtecan (Enhertu) from August 2024 to February 2025 resulted in a partial response, but disease eventually progressed.

By this point, the patient was severely symptomatic with dyspnea (Eastern Cooperative Oncology Group 3) with extensive bilateral lung metastases, requiring long-term oxygen support. She became wheelchair-bound. Tisotumab vedotin was not available or licensed locally at that time.

**FIG 1.**
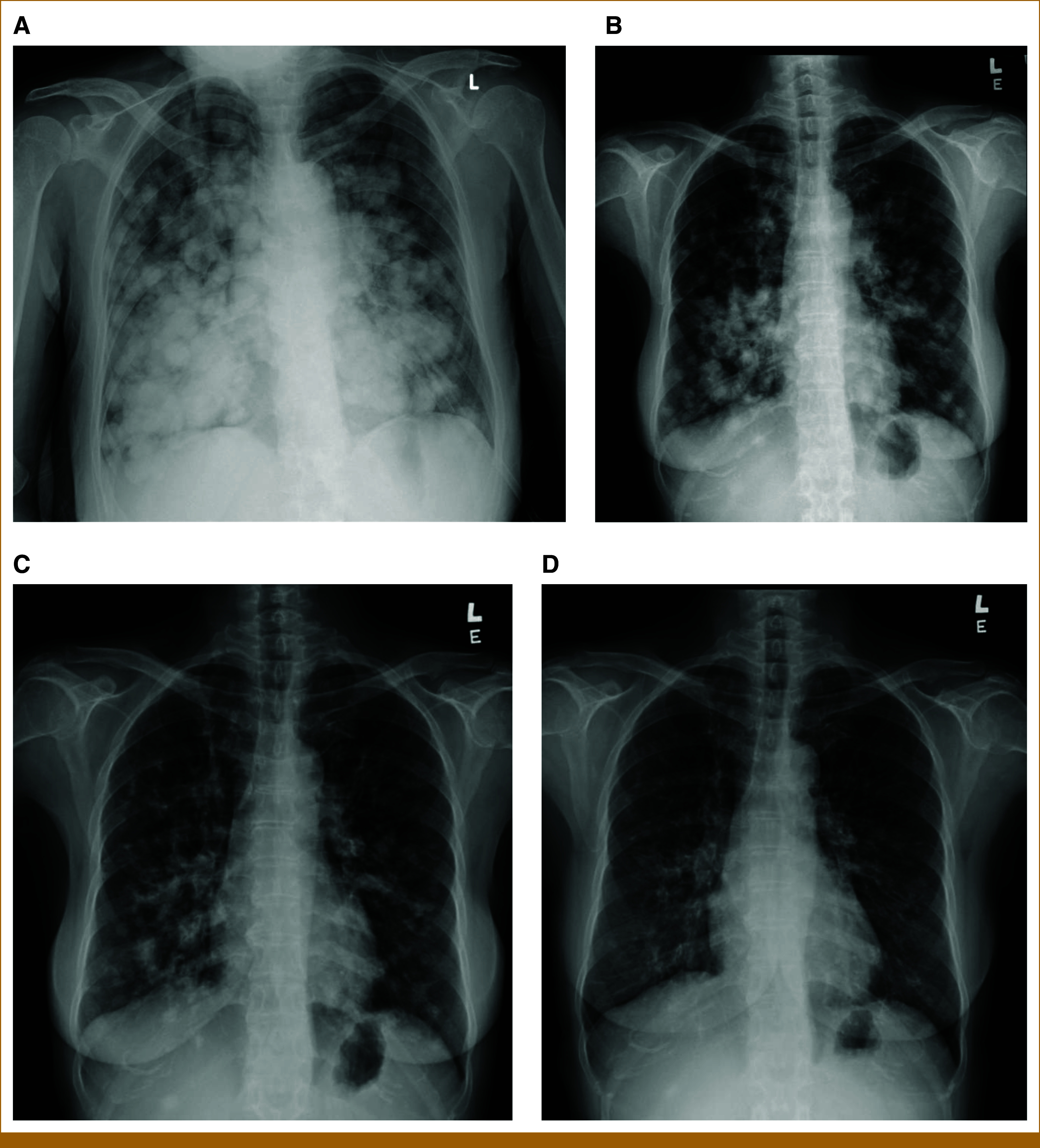
(A) CXR before the start of sotorasib. (B) CXR 1 week after the start of sotorasib. (C) CXR 2 weeks after the start of sotorasib. (D) CXR 6 months after the start of sotorasib.

The patient was initiated on sotorasib at 480 mg once daily (half the usual daily dose) on March 13, 2025, because of poor performance status at that time. Remarkable improvement was observed 1 week after starting sotorasib, with resolution of dyspnea and discontinuation of supplemental oxygen. ECOG functional status improved to 1. She became fully ambulatory. Following significant improvement in functional status, the dose was escalated to the full 960 mg once daily on April 10, 2025. Serial chest X-rays showed marked reduction in bilateral lung metastases (Figs 1A-1D). However, a dose reduction to 720 mg once daily has been required since July 10, 2025, because of the development of grade 2 thrombocytopenia. Apart from thrombocytopenia, no other adverse events were reported.

**FIG 2.**
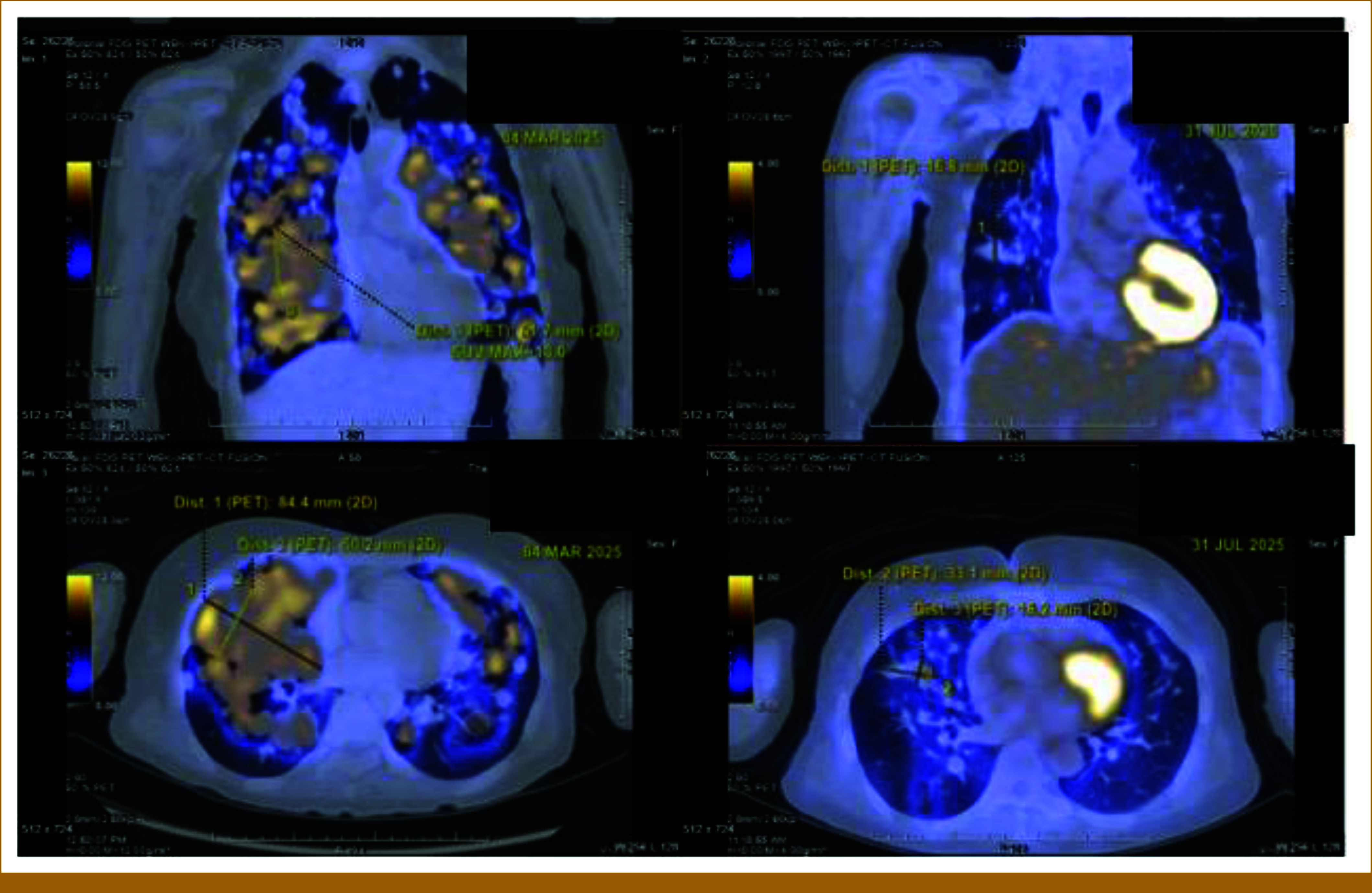
PET scan before (left) and 4 months after treatment of sotorasib (right). PET, positron emission tomography.

A positron emission tomography-computed tomography performed at 4 months post–sotorasib treatment demonstrated stable brain metastases with no new lesions, completely resolved mediastinal lymphadenopathy, and significantly reduced number, size, and metabolic activity of bilateral lung metastases, consistent with an almost complete metabolic response (Fig 2). The CXR 6 months after treatment showed no progression still.

The patient has maintained clinical benefit and improved quality of life for at least 6 months, with no disease-related complications or significant toxicity to date. Written informed consent for publication was obtained from the patient.

## Discussion

Mesonephric carcinoma of the cervix is exceptionally rare arising from the mesonephric remnant of the gynecologic tract. It primarily affects the uterine cervix, with rare occurrences in the uterine corpus, ovaries, and vagina. It may develop from Müllerian tissue displaying mesonephric differentiation or from mesonephric remnants within the uterine wall. It is an aggressive malignancy, with limited treatment options in advanced or refractory stages. At a molecular level, it is frequently characterized by *KRAS* mutations, which serve as key oncogenic drivers in the tumor's pathogenesis. This case reinforces the importance of understanding the molecular landscape of rare cancers to guide treatment decisions.

A case series of seven mesonephric carcinomas of female genital tract showed 100% *KRAS* mutation (four G12D and three G12V).^1^ Another series reported eight cases of mesonephric carcinoma with a prevalence of *KRAS* mutation of 85%; however, only around 6%-7% harbors the specific G12C mutation.^2^ A Chinese series of 17 patients reported nine of 10 patients who had genetic testing that showed *KRAS* mutation.^3^ In a broader sense across diverse tumour histologies, a retrospective series of more than 3,000 patients with rare tumors demonstrated prevalence of *KRAS* mutation of around 8% overall and variable prevalence of *KRAS* G12C mutation, with higher frequencies in sarcomatoid lung carcinoma (5.7%) and clear cell ovarian cancer (5.4%), but it was absent in small-bowel cancers.^4^

KRAS proteins act as a molecular switch, tightly regulating various signaling pathways by transitioning between active and inactive states. Once activated, *KRAS* triggers multiple signaling pathways, including the RAF-MEK-ERK pathway, the PI3K-AKT-mTOR pathway, and others, demonstrating their extensive role in coordinating diverse cellular signaling networks.^5^

Targeting the *KRAS* pathway through the combination of avutometinib and defactinib has emerged as an effective strategy for adult patients with recurrent, *KRAS*-mutated low-grade serous ovarian cancer (LGSOC) who have received prior therapy. In the phase I FRAME trial^6^ involving patients with LGSOC, the objective response rate (ORR) was 42.3% (95% CI, 23.4 to 63.1), with a median progression-free survival (PFS) of 20.1 months (95% CI, 11.2 to 43.9). In the phase II RAMP-201 trial,^7^ this dual-inhibition approach demonstrated efficacy in the *KRAS*-mutant LGSOC cohort, with a confirmed ORR of 44% and a median PFS of 22 months (95% CI, 11.1 to 36.6).

*KRAS*-G12C–targeted therapies, such as sotorasib, have shown promising response data in other *KRAS*-mutant malignancies, particularly non–small cell lung cancer (NSCLC) and pancreatic and colorectal cancers. The pivotal phase II CodeBreaK 100 trial demonstrated a disease control rate of up to 80% and a PFS rate of up to 7 months in patients with *KRAS* G12C–mutated NSCLC who progressed on previous standard therapies,^8^ as well as significant anticancer activity with an acceptable safety profile in previously treated *KRAS* G12C–mutated advanced pancreatic cancer.^9^ The phase III CodeBreaK 300 trial showed that, in patients with refractory colorectal cancers who progressed on previous standard therapies, sotorasib combined with panitumumab resulted in an ORR of 26% and a hazard ratio of almost 0.5 compared with standard therapies.^10^ Other *KRAS* G12C inhibitors, like adagrasib,^11^ also showed consistent benefit for patients with NSCLC. These findings have underscored the potential of KRAS inhibition in cancers where this mutation plays a pivotal role.

In this patient, the identification of a *KRAS* G12C mutation through NGS was critical in informing the treatment strategy and enabling the use of targeted therapy.

This case highlights the importance of comprehensive molecular profiling, particularly in rare and treatment-resistant cancers, where conventional therapies often fail. The marked clinical and radiologic response to sotorasib in this case demonstrates its efficacy in *KRAS*-driven mesonephric cervical carcinoma, further establishing the role of precision oncology in managing rare malignancies.

This case adds to the growing evidence supporting the utility of molecularly targeted therapies in improving outcomes for patients with heavily pretreated, advanced-stage cancers. It expands the clinical evidence for sotorasib beyond common cancers, demonstrating its potential in rare tumor types, and underscores the value of precision medicine in improving outcomes for aggressive malignancies with limited treatment options.

In conclusion, this case highlights the importance of genomic testing in mesonephric tumors and showed, to our knowledge, for the first time, clearly significant clinical and radiologic efficacy of *KRAS* G12C inhibitors, such as sotorasib, in heavily pretreated metastatic mesonephric cervical cancer harboring this specific mutation. Even in the setting of severe disease burden, chemotherapy refractoriness, and poor functional status, sotorasib provided rapid, durable, and significant clinical and radiologic improvement with minimal toxicities. Molecularly targeted therapy offers a promising option for managing rare and aggressive malignancies like mesonephric cervical cancer.
